# Brain‐Computer Interface: Bring Care Into a Future Phase? Challenges and Opportunities for Nursing in the Era of Emerging Technologies

**DOI:** 10.1002/nop2.70345

**Published:** 2025-11-02

**Authors:** Quanlei Li, Edmond Pui Hang Choi, Maling Gou, Yali Tian, Diana Baptiste

**Affiliations:** ^1^ School of Nursing, Li Ka Shing Faculty of Medicine, The University of Hong Kong, Pokfulam Hong Kong SAR China; ^2^ Department of Biotherapy, Cancer Center and State Key Laboratory of Biotherapy, West China Hospital Sichuan University Chengdu China; ^3^ West China Hospital/West China School of Nursing Sichuan University Chengdu China; ^4^ School of Nursing Johns Hopkins University Baltimore Maryland USA

**Keywords:** brain‐computer interfaces, digital health, health equity, health technology, nursing, nursing ethics

## 1 Introduction

When Florence Nightingale founded modern nursing, she emphasised observation, sanitation, compassion and scientific reasoning. Her vision of nursing as a science, an art and a social responsibility continues to inspire the profession. More than a century later, nursing is entering an era shaped by rapid technological change. Artificial intelligence (AI), robotics and neurotechnology are reshaping how humans live, suffer and recover. Among these, brain‐computer interface (BCI) has emerged as a powerful tool capable of translating brain activity into digital signals, enabling new forms of interaction between individuals and their environments.

The International Council of Nurses (ICN) recently renewed its definition of nursing, highlighting health equity, cultural safety and people‐centered care as central values. This definition underscores the leadership role of nurses in shaping research, policy, education and healthcare systems. Importantly, it situates nurses as key actors in ensuring that technological innovations, including BCI, contribute to equitable health outcomes rather than exacerbate disparities.

## 2 A Brief Overview of Brain‐Computer Interface

BCI refers to systems that record and convert brain activity into commands for external devices, allowing individuals to interact with the world without muscle involvement. The concept was first introduced by Jacques Vidal in the 1970s through electroencephalography (EEG)‐based computer input. Early research focused on supporting patients with severe motor disorders, while advances in signal acquisition and processing have enabled real‐time applications in clinical care (Liu et al. 2025).

BCI technologies are typically categorised into three types. Non‐invasive BCI systems, such as EEG‐based systems, collect brain signals through electrodes placed on the scalp. They are safe, affordable and increasingly applied in communication support, motor rehabilitation, and neurofeedback training. Invasive BCI systems use implanted electrodes to provide high‐resolution signals but involve significant surgical and ethical risks, limiting their widespread clinical use. Hybrid BCI systems combine EEG with other signals (e.g., electromyography or eye movements) to enhance accuracy and stability. Each approach offers unique advantages and limitations in terms of accuracy, safety and clinical integration. For nursing practice, non‐invasive EEG‐based BCI currently presents the most feasible path for integration into patient care, while invasive and hybrid systems remain primarily experimental.

## 3 Clinical Applications and Nursing Integration

Over the past two decades, BCI has gradually transitioned from laboratory research to clinical use, offering new ways to restore communication, enhance rehabilitation, support cognitive and emotional well‐being.

In neurological rehabilitation, EEG‐based BCI training has been incorporated into the therapeutic protocols of stroke patients, particularly those with upper limb impairments in subacute or chronic phases. A recent meta‐analysis of 21 randomised controlled trials involving 886 patients confirmed that when BCI training is combined with functional electrical stimulation or motor imagery, it can significantly improve patients' upper limb motor recovery (Li et al. 2025). Such evidence provides nurses with opportunities to design individualised and rehabilitation‐focused care strategies that integrate BCI technologies.

In the context of critical care and communication, Eliseyev et al. (2021) reported that over one third of conscious intensive care unit (ICU) patients and all healthy volunteers successfully operated a self‐paced BCI system, despite the challenges of a complex and noisy ICU environment. This application highlights BCI's potential to safeguard patient dignity and ensure equitable access to care by reducing communication barriers. Nurses are uniquely positioned to guide these interventions through patient engagement, care coordination and ethical oversight.

In gerontological care and cognitive support, a recent systematic review of 16 studies reported that BCI and neurofeedback technologies may enhance cognitive functions such as working memory and attention among older adults (Tsai et al. 2025). Although evidence quality remains variable, these findings offer early support for BCI as a non‐pharmacological strategy for supporting aging populations.

Emerging research also shows that EEG‐based BCI systems can help identify internal emotional states, particularly in situations where verbal communication is limited or where social desirability bias may compromise the accuracy of self‐reported information. Preliminary findings have revealed associations between neural activity patterns and emotional responses, although accuracy rates vary (Kanna et al. 2025). Evidence indicates that BCI technologies can support real‐time emotion detection in care delivery, providing nurses with new opportunities to identify patients' unexpressed distress, concealed pain, undisclosed violence and socially desirable response patterns during sensitive interactions.

The integration of AI technology has further expanded the potential applications of BCI in clinical settings. Advanced AI algorithms have been increasingly used to decode complex neural signals in real time, improving the response speed and accuracy of emotion and intent detection (Liu et al. 2025). Such synergy between BCI and AI enables individuals to operate assistive devices, such as robotic limbs, communication interfaces or mobility aids, through cognitive intent, providing a further practical foundation for the medical application of BCI.

As BCI becomes increasingly integrated into healthcare systems, nurses and other healthcare professionals need to develop a basic understanding of its structure, functionality and ethical issues. Such knowledge not only supports the safe and informed use of BCI technologies during clinical practice, but also enables nurses to actively participate in the design, evaluation and optimization of emerging technologies. Nurses should play a key role in the application of emerging technologies, and ensure that these technologies are consistent with people‐centered values, care standards and health priorities.

The World Health Organization's Global Strategy on Digital Health 2020–2025 clearly states that health workers should possess core competencies related to digital technology and AI in order to promote the continued strengthening of healthcare systems. The State of the World's Nursing Report 2025 further emphasises the need to increase investment in digital literacy, technological leadership and professional development to support nurses in playing an increasingly important role in promoting equitable and resilient healthcare systems. Despite growing global interest in digital transformation, BCI remains extremely limited in nursing education, clinical guidelines, and mainstream nursing research. To date, there are no published studies that explicitly explore the specific roles of nurses in the development, implementation or evaluation of BCI systems.

## 4 Implementation Challenges and Strategic Directions

Preliminary research and practical examples have shown promise; however, the integration of BCI into routine clinical practice remains limited. Several key challenges continue to hinder its meaningful and effective applications, particularly in the context of nursing care delivery.

Technical challenges remain the core difficulty during clinical application. These include unstable signal acquisition, limited classification accuracy and insufficient device usability in real‐world clinical environments, all of which hinder BCI implementation. Currently, most BCI systems still need to be operated under highly controlled laboratory conditions and rely on the support of technical personnel, severely limiting their accessibility in typical healthcare settings.

Ethical issues regarding patient autonomy, privacy protection, and informed consent remain unresolved. As BCI systems become increasingly complex and merge with AI, the interpretability of algorithmic decision‐making becomes increasingly critical and challenging. As frontline advocates for patient rights, nurses must approach these ethical issues and even dilemmas with a high degree of sensitivity, ensuring that patients' rights and dignity remain at the core of technology‐driven interventions.

Equity must remain a central consideration. Without deliberate attention to equitable access, BCI technologies may remain confined to high‐resource settings, thereby exacerbating global health disparities. Nursing, as the discipline most closely engaged with patients across diverse care contexts, is well positioned to advocate for and implement BCI technologies that are accessible in both high‐ and limited‐resource settings. Achieving such equity, however, may prove as challenging as overcoming BCI technological barriers themselves.

Interdisciplinary collaboration has long been a challenge and is expected to continue in the future. Existing research has rarely explored how nurses participate in the use of BCI in their daily practice, including device management, patient communication and care planning. This lack of attention partially reflects the lack of interdisciplinary collaboration between engineers, neuroscientists, ethicists, nurses and other key stakeholders. Without coordinated efforts to establish a shared framework, nursing perspectives will continue to be marginalised in the development and clinical integration of BCI.

## 5 Nursing in a Digitally Mediated Future

Global health faces urgent challenges, including aging populations, chronic diseases and workforce shortages. In this context, nurses are increasingly recognised as leaders in policy advocacy, health promotion and system transformation. Nursing education should expand to include digital literacy and competencies in emerging technologies, enabling nurses to critically evaluate and guide the use of BCI. Professional development initiatives must foster interdisciplinary collaboration, ensuring that nurses can influence design, implementation and evaluation processes. Nurses have a responsibility to ensure that BCI is developed and applied in ways that advance the ICN's vision of health equity and people‐centered care.

As Nightingale's lamp once symbolised compassion and science amid suffering, BCI may now serve as a beacon of technological and humanistic integration. With careful guidance, nursing can ensure that the digital future of health care remains grounded in equity, dignity and compassion. Nursing scholars and practitioners must not only participate in the technological development of BCI, but also lead its ethical, educational and policy directions, ensuring that technologies ultimately strengthen health equity and the humanistic essence of care.

## Conflicts of Interest

The authors declare no conflicts of interest.

## Data Availability

The authors have nothing to report.
